# Potential for detecting a monopole-like topological magnetoelectric response using image potential states

**DOI:** 10.1093/nsr/nwag548

**Published:** 2026-08-26

**Authors:** Yang Zhan, Huaiyu Zhang, Yu Wang, Chenmin Liu, Jian-Qiang Zhong, Yeliang Wang, Hong-Jun Gao, Yi-Qi Zhang, Baojie Feng, Peng Cheng, Lan Chen

**Affiliations:** Institute of Physics, Chinese Academy of Sciences, Beijing 100190, China; School of Physical Sciences, University of Chinese Academy of Sciences, Beijing 101408, China; Institute of Physics, Chinese Academy of Sciences, Beijing 100190, China; School of Physical Sciences, University of Chinese Academy of Sciences, Beijing 101408, China; Institute of Physics, Chinese Academy of Sciences, Beijing 100190, China; School of Physical Sciences, University of Chinese Academy of Sciences, Beijing 101408, China; Institute of Physics, Chinese Academy of Sciences, Beijing 100190, China; School of Physical Sciences, University of Chinese Academy of Sciences, Beijing 101408, China; School of Physics, Hangzhou Normal University, Hangzhou 311121, China; MIIT Key Laboratory for Low-Dimensional Quantum Structure and Devices, School of Integrated Circuits and Electronics, Beijing Institute of Technology, Beijing 100081, China; Institute of Physics, Chinese Academy of Sciences, Beijing 100190, China; School of Physical Sciences, University of Chinese Academy of Sciences, Beijing 101408, China; Institute of Physics, Chinese Academy of Sciences, Beijing 100190, China; School of Physical Sciences, University of Chinese Academy of Sciences, Beijing 101408, China; Institute of Physics, Chinese Academy of Sciences, Beijing 100190, China; School of Physical Sciences, University of Chinese Academy of Sciences, Beijing 101408, China; Institute of Physics, Chinese Academy of Sciences, Beijing 100190, China; School of Physical Sciences, University of Chinese Academy of Sciences, Beijing 101408, China; Institute of Physics, Chinese Academy of Sciences, Beijing 100190, China; School of Physical Sciences, University of Chinese Academy of Sciences, Beijing 101408, China

**Keywords:** image magnetic monopole, topological magnetoelectric effect, scanning tunneling microscopy, image potential states

## Abstract

Magnetic monopoles, hypothetical particles behaving as isolated magnetic charges, have long been predicted by theories beyond the standard model but remain experimentally elusive. Qi *et al*. proposed that magnetic monopoles could be realized in real space by applying an active electric field at a topological-insulator/vacuum interface. Here, we use field-emission scanning tunneling microscopy to investigate image potential states (IPSs) on higher-order topological insulator Bi(111) and observe anomalous IPS splitting. By varying substrate dielectric properties and film thickness, we find that the splitting correlates with the radial active electric field and the emergence of Bi(111) topological surface states. These observations are compatible with a phenomenological scenario in which the active radial electric field couples to the Bi (111) surface states and induces an effective monopole-like topological magnetoelectric response. Our results establish field-emission IPS spectroscopy as a sensitive platform for probing active-field-induced magnetoelectric responses at topological surfaces.

## Teaser

Anomalous splitting of image-potential states on HOTI Bi(111) provides a new spectroscopic window into possible monopole-like topological magnetoelectric responses.

## INTRODUCTION

The electromagnetic response of conventional insulators is linear. However, Qi *et al*. [1] have proposed that topological insulators (TIs) can host a nonlinear magnetoelectric response, known as the topological magnetoelectric effect (TME). The effective action *S_θ_* can be written as


(1) \begin{eqnarray*}
{S}_\theta = \frac{\alpha \theta} {{4\pi}^2} \int {\rm {d}^3} x{\rm d}t\, {\boldsymbol {E \cdot B}},
\end{eqnarray*}

where ${{\mathrm{d}}}^3x{\mathrm{d}}t\ $is the volume element of space and time, ***E*** and ***B*** are the electric and magnetic fields defined within an insulator, *α * is defined as ${{\mathrm{e}}}^2/\hbar {\mathrm{c}}$, whereas *θ* = 0 for conventional insulators and *θ* = π for TIs. Previous theoretical [2,3] and experimental [4–7] studies have attempted to introduce ferromagnetic or antiferromagnetic order into TIs, thereby transforming the system into either quantum anomalous Hall insulators or axion insulators, which manifest as integer and zero Hall conductivity plateaus in transport measurements, respectively. These works support the validity of the TME and indirectly suggest that electric and magnetic fields may be commutative in specified condensed matter systems. More importantly, it is implied that, by symmetry, the existence of electric monopoles would permit the existence of magnetic monopoles in principle, but has not been experimentally realized.

Building on the TME effect, a theoretical proposal has been put forward to construct magnetic monopoles in real space [8]. The equivalent active magnetic field can be generated by applying an active electric field ($\nabla \cdot E \not= 0$) to a symmetry-broken surface (e.g. the TI-vacuum interface). Experimentally, the field-emission mode of a scanning tunneling microscope (STM) offers a direct method to produce such an active-electric-field geometry, in which electrons are emitted from the tip and injected into the sample by applying a voltage exceeding the work function of the metallic tip. The attractive potential between the electron and its image charge can give rise to bound states known as image potential states (IPSs), which have been well studied by STM [9–14].

Previous studies of IPSs on the surface of three-dimensional (3D) TIs (Bi_2_Te_3_ [15] and Sb [16]) revealed that the dissipation mechanism and lifetime of IPSs are influenced by the topological surface states. However, investigations of IPSs on the surface of higher-order topological insulators (HOTIs) are absent. Bismuth is the heaviest element among stable elements, and its exceptionally strong spin–orbit coupling induces band inversion, unconventional topological states [17–21], and unique transport properties such as anomalous Hall [22,23] and nonlinear Hall response under zero magnetic field [24], making it an attractive platform for studying the interaction between active electric fields and topological surface states. In this work, we measured IPSs on Bi (111) thin films with varying thicknesses by STM, and discovered an anomalous peak-splitting phenomenon in samples beyond three bilayers. By switching the film on the substrates with different dielectric constants to alter the distribution of the electric field from the tip, the splitting of IPSs can be correlated with the radial component of the electric field. These results indicate that field-emission IPS spectroscopy provides a practical platform for examining how an active radial electric field interacts with the development of Bi (111) surface states. Within a phenomenological framework, the observed splitting can be discussed in terms of an effective magnetic response associated with a monopole-like TME scenario. In this sense, the present work suggests a possible spectroscopic connection between field-emission IPS spectroscopy and the active-field geometry envisioned in the Qi model.

## RESULTS

### IPSs on Bi(111)/Si(111) heterojunction

We first deposited bismuth (Bi) atoms on Si(111) to saturate the dangling bonds of Si atoms, forming a $\sqrt{3} \times \sqrt{3} $ reconstruction referred to as β-Bi [25]. Further deposition of Bi atoms on this reconstructed surface resulted in the formation of Bi(110) islands with a black phosphorus-like structure. Once the film thickness exceeded 4 bilayers (BL), Bi(110) underwent a structural phase transition into a Bi(111) structure [26]. In our experiments, we fabricated a high-quality Bi(111) crystalline thin film with a thickness over 10 BL on the Si(111) surface, as shown in Fig. 1a. The atomic-resolution STM images of the Bi(111) surface reveal a hexagonal close-packed arrangement with a lattice constant of *a* = 4.5 Å (Fig. 1b). Scanning tunneling spectroscopy (STS) measurements in Fig. 1c taken on the terrace exhibit a characteristic peak at 220 meV, while those near the edge exhibit a peak at 180 meV, corresponding to the van Hove singularities of the surface and edge states, respectively. Figure 1d and e, recorded synchronously at 180 mV, shows the topography and local density of states of a triangular Bi island, demonstrating that the peak is highly localized at the island’s edge. The observed terrace and edge spectral features are consistent with the characteristic surface and edge electronic structure reported for HOTI Bi(111) [18,19].

**Figure 1.**
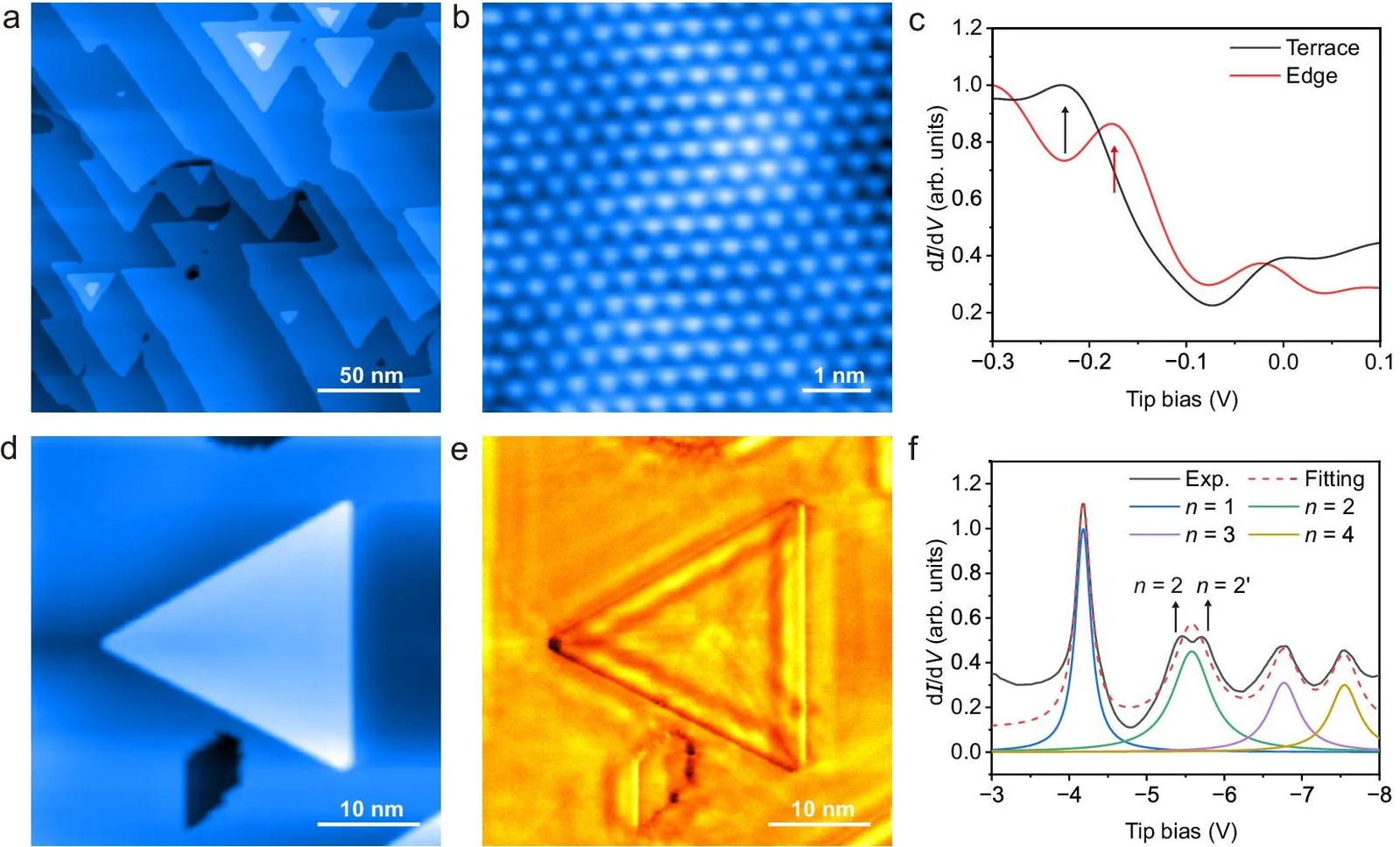
IPSs on HOTI Bi(111)/Si(111) thin film. (a) Topographic image of 10-BL Bi (111) thin film on Si(111) substrate (200 × 200 nm^2^). (b) Atomically resolved STM topographic image (6 × 6 nm^2^). (c) STS on the terrace away from the edges and near the edge (setpoint: *V*_t_ = −0.3 V, *I*_t_ = 100 pA). Arrows indicate the vHS of surface states and edge states, respectively. (d) Topographic image of one triangular Bi(111) island. (e) d*I*/d*V* mapping simultaneously acquired with the topographic image (d) (setpoint: *V*_t_ = −180 mV, *I*_t_ = 100 pA), showing high conductance at each edge of the triangular island. (f) IPSs measured on the terrace away from the edges (setpoint: *V*_t_ = −2 V, *I*_t_ = 200 pA). Fitting the four IPSs using Lorentzian distributions reveals that only the *n* = 1 IPS peak exhibits good agreement with Lorentzian statistics. The *n* = 2 IPS splits into two sublevels 2/2′, while the *n* = 3/4 IPSs display asymmetric characteristics, significantly deviating from Lorentzian statistics.

We then increased the bias above the tip work function, causing electrons to be emitted from the tip apex through field emission, inducing image charges below the sample surface. Perpendicular to the sample surface, the field-emitted electron resides in a *- 1/z* potential well formed by its image charge, resulting in a series of bound-states known as IPSs [16]. If the bias voltage matches the energy of an IPS, then the transmission probability and differential conductance increase abruptly, corresponding to multiple discrete IPS peaks in STS. First, we measured standard IPSs spectrum on Si(111) and β-Bi, which are topologically trivial, revealing multiple independent IPS peaks shown in Fig. S1. The shape of each peak follows a Lorentzian distribution. According to the central limit theorem, the superposition of multiple Lorentz peaks with varied central energies converges toward Gaussian statistics. Therefore, our fitting results for each IPS peak on the Si and β-Bi surfaces accordingly closely follow a Gaussian distribution (Fig. S1). The full width at half maximum (FWHM) of each IPS peak exhibits a decreasing trend as energy increases, indicating that IPS lifetimes increase with increasing principal quantum number [16,27].

In contrast to the topologically trivial samples, the IPS spectra on the surface of Bi(111) thin films (Fig. 1f) show that only the *n* = 1 IPS exhibits a standard Lorentzian line shape, while the *n* = 2 IPS anomalously splits into two distinct subpeaks. The *n* = 3 and 4 IPSs also display asymmetry, deviate from both Lorentzian and Gaussian distributions, and possess a markedly larger FWHM than the *n* = 1 level, suggesting that IPS peaks with higher quantum numbers (≥3) may also be split, although the subpeaks are hardly distinguished due to broadening. When the energy lies above the sample work function, the electronic states are generally expected to be extended rather than form new bound-state levels. This makes it unlikely that the additional split peak arises from an independent bound state, and instead suggests that it is associated with the IPSs.

Intrinsic IPSs can also be measured using two-photon photoemission (2PPE) [10,28–30]. As no externally applied electric field is required, the 2PPE experiments provide an intrinsic representation of the IPS energy levels [12]:


(2) \begin{eqnarray*}
{E}_n = {E}_{{\mathrm{vac}}} - \frac{{0.85\ {\mathrm{e}}V}}{{{{\left( {n + a} \right)}}^2}},\ n = 1,2,...
\end{eqnarray*}

where *E*_vac_ is the vacuum energy level, and *a* is a crystal-field correction factor. The first three intrinsic IPSs on the surface of bismuth measured by 2PPE occur at *E* = −3.571, −4.052 and −4.170 [28]. On the other hand, in STM measurements, the metallic tip introduces an extremely strong electric field of ∼10^9^ V m^−1^ in the tunnel junction. Similar to the Stark shift of a Shockley surface state under a strong external electric field [31], IPSs also exhibit a comparable shift. Moreover, since the IPS wave functions are exposed beyond the lattice periodic potential, they are affected more intensely by the electric field [9,16,32]. Therefore, the IPSs in Fig. 1f shift overall toward higher energy levels compared with the 2PPE results due to the Stark effect. Furthermore, the *n* = 2 IPS measured in the 2PPE experiments does not split [28]. We speculate that the peak splitting of IPSs in the STM measurements originates from the tip electric field.

During the field emission process in STM, the electric field distribution at the tunneling junction consists of two components: one originating from the metallic tip and the other provided by the field-emitted electrons. For the tip, electrons tend to concentrate at the point of greatest curvature (the tip apex), which can be simplified as a point charge q. Using the mirror charge method to estimate the electric field generated by the tip, we consider an induced image charge with ${\rm q}^{\prime}\ = \frac{{{\epsilon }_r - 1}}{{{\epsilon }_r + 1}}{\rm q}$ (${\epsilon }_r$ is the relative permittivity of the sample) within the dielectric material. The sample surface is subjected to a vertical electric field (*E_z_*) and a horizontally distributed electric field (*E_r_*) with central symmetry, which are, respectively, expressed as


(3) \begin{eqnarray*}
{E}_{z,{\mathrm{tip}}}\left( {z = 0} \right) = \frac{\rm q}{{4\pi {\epsilon }_0}} \cdot \frac{{2{\epsilon }_r}}{{{\epsilon }_r + 1}} \cdot \frac{1}{{{d}^2}},
\end{eqnarray*}

(4) \begin{eqnarray*}
{E}_{r,{\mathrm{tip}}}\left( {z = 0} \right) = \frac{\rm q}{{4\pi {\epsilon }_0}} \cdot \frac{2}{{{\epsilon }_r + 1}} \cdot \frac{r}{{{{\left( {{d}^2 + {r}^2} \right)}}^{\frac{3}{2}}}},
\end{eqnarray*}

where ${\epsilon }_0$ is the vacuum permittivity, *d* is the tip–sample distance, and *r* is the distance from the center of charge q. The magnitude of this electric field is determined by the dielectric permittivity ${\epsilon }_r$ and the distance *d* to the sample. Further considering electron field emission as a dynamical process, a single electron emitted from the tip accelerates through the tunneling junction (about 1 nm) under the influence of a strong electric field (10^9^ V m^−1^) with a travel time estimated to be about 1 femtosecond (fs). Then the electron occupies an IPS for several femtoseconds [16], and finally the current forms from the IPSs relaxing into the sample. For a semiconductor with a typical carrier concentration on the order of 10^18^ cm^−3^, the plasmon frequency ${\omega }_{\mathrm{p}} = \sqrt {\frac{{n{{\mathrm{e}}}^2}}{{{\epsilon }_0{m}^*}}} $ is about 10^14^ s^−1^. We estimate the time required to establish electrostatic equilibrium is on the order of 100 fs, much greater than the electron traveling time across the junction. Therefore, the electric field from field-emitted electrons is contributed entirely by themselves. When the emitted electron is at a distance *d*_e_ from the sample, the instantaneous electric field on the sample surface can be expressed as: ${E}_{z, {\mathrm{e}}}( {z = 0} ) = \frac{\rm e}{{4\pi {\epsilon }_0}} \cdot \frac{1}{{d_{\mathrm{e}}^2}}$ and ${E}_{r,\ {\mathrm{e}}}( {z = 0} ) = \frac{\rm e}{{4\pi {\epsilon }_0}} \cdot \frac{r}{{{{( {d_{\mathrm{e}}^2 + {r}^2} )}}^{3/2}}}$, which depend only on the orbital radius and lifetime of the IPSs. Both radial electric field *E_r_* generated by metallic tip and field-emitted electrons can be regarded as an active electric field within the *x–y* plane with $\nabla \cdot {E}_r \not= 0$.

The carrier concentration of bulk bismuth is comparable to typical semiconductors (10^17^ cm^−3^) [33,34], while our grown Bi(111) film with 4 nm thickness lacks sufficient free carriers to establish electrostatic equilibrium under strong external electric field. Consequently, the distribution of the electric field within the tunnel junction is primarily determined by the dielectric properties of the substrate. Considering Si(111) is a typical semiconductor substrate with a dielectric constant ${\epsilon }_r$ = 11.7, we give the schematic diagram of the electric field distribution in STM tunnel junction shown in Fig. 2a. As the tunneling current increases, the tip-sample distance decreases while the tip electric field increases simultaneously, pushing all IPSs to higher energies due to Stark effects under stronger fields, as shown in Fig. 2b. We summarize the relationship between the tunneling current and the energy of IPSs in Fig. 2c, and the dependence of the splitting energy between *n* = 2/2′ IPS on the tunneling current in Fig. 2d. The splitting magnitude gradually increases from 0.28 to 0.55 eV as the tunnel junction distance decreases and the tip electric field increases. This trend is similar to the Stark shift, suggesting that the splitting also originates from the tip electric field.

**Figure 2.**
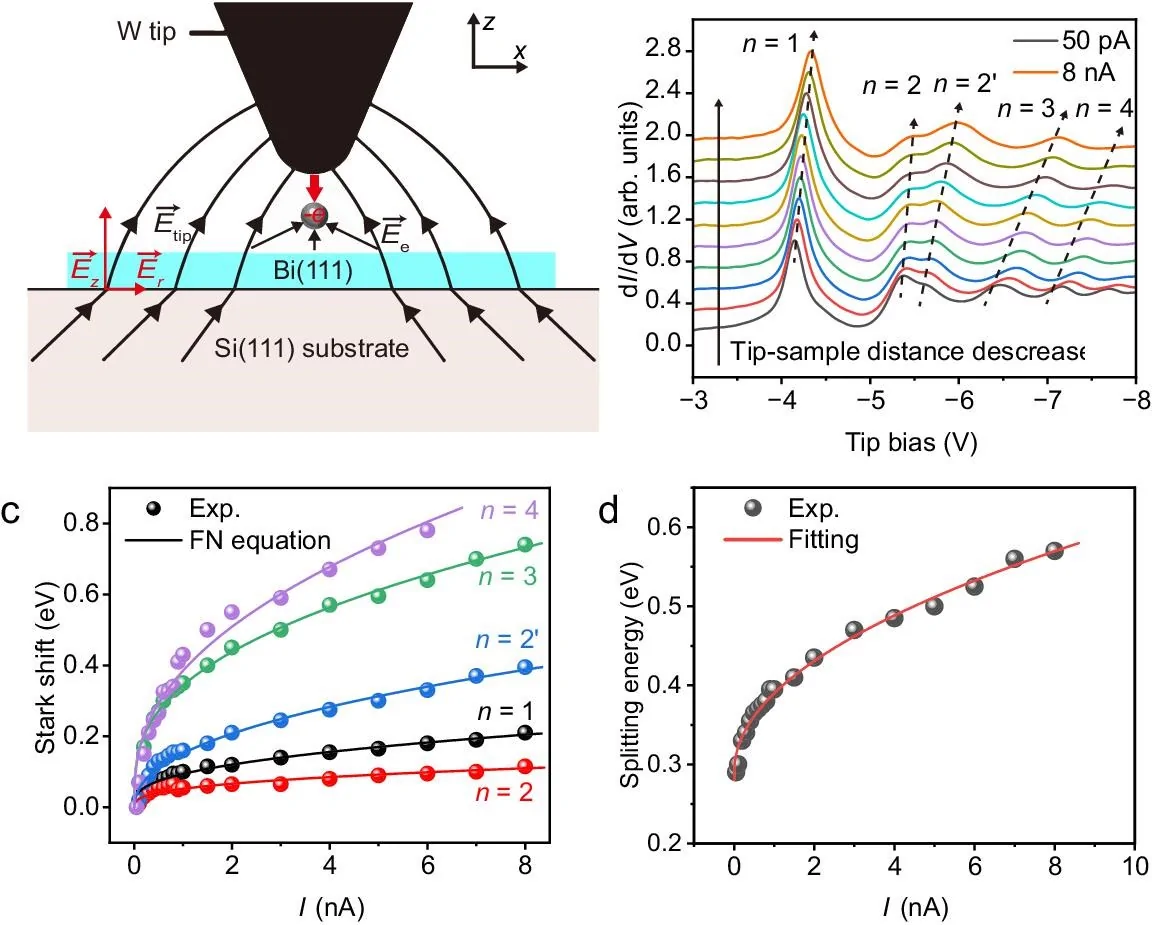
Electric field dependence of IPSs on Bi(111)/Si(111). (a) Schematic diagram of the electric field distribution in a metal–vacuum–semiconductor tunnel junction. Metallic tip and semiconductor substrate achieve electrostatic equilibrium, contributing to a radial active electric field (${E}_r \not= 0$). The emitted electrons also contribute an instantaneous active electric field. (b) IPSs recorded at different setpoints (*V*_t_ = −2 V, *I*_t_ = 50 pA–8.0 nA). Each spectral line is vertically offset by 0.1 (arb. units). (c) Energy shifts of IPSs with tunneling current due to the Stark effect. (d) The splitting energy between *n* = 2/2′ IPS subpeaks varies with the tunneling current. The splitting energy continuously increases from 0.28 to 0.55 eV. The points in (c) and (d) were fitted using the Fowler–Nordheim (FN) equation.

### IPSs on Bi(111)/Sb(111) heterojunction

To further investigate the splitting behavior of IPSs in the electric field, we examined the IPSs of Bi(111) on a metallic substrate. Then we prepared high-quality Bi(111) films with thicknesses ranging from 1 to 10 BL on Sb(111) substrate. Figure 3a shows a large-area Bi(111) film grown on Sb(111) with the same thickness as on Si(111), and the atomic-resolution image displays a lattice constant of *a* = 4.4 Å (Fig. 3b), which is ∼2% compressive strain compared with that on Si(111). Figure 3c–e presents the characterization of the topological surface states and edge states of Bi(111) on Sb(111) substrate through STS and d*I*/d*V* mapping, indicating that the topological properties of the film remain unchanged under substrate-induced stress. Figure 3f displays the IPS peaks of Bi(111) films with different thicknesses on Sb(111). For Bi(111) films with thicknesses of 1–2 bilayers, several distinct IPS peaks corresponding to principal quantum numbers *n* occur, similar to the situation on the topologically trivial semiconductors shown in Fig. S1. Once the film thickness exceeds three bilayers, the IPS undergoes a significant splitting and subsequently persists up to ten bilayers or beyond.

**Figure 3.**
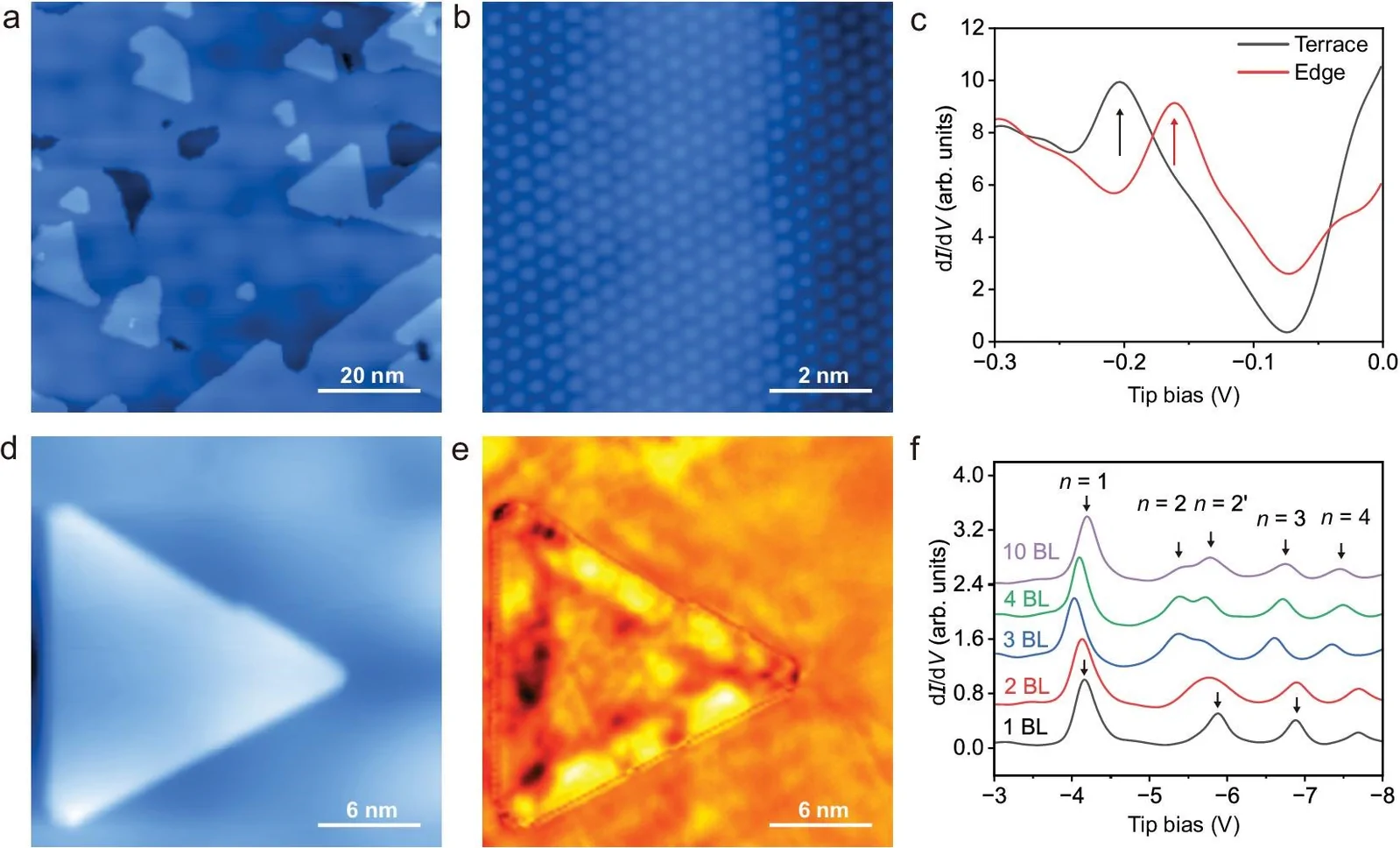
IPSs on HOTI Bi(111)/Sb(111) thin film. (a) Topographic image of 10-BL Bi(111) thin film on Sb(111) substrate (80 × 80 nm^2^). (b) Atomically resolved STM topographic image (8 × 8 nm^2^). (c) STS on the terrace away from the edges and near the edge (setpoint: *V*_t_ = −0.3 V, *I*_t_ = 100 pA). Arrows indicate the vHS of surface states and edge states, respectively. (d) Topographic image of one triangular Bi(111) island. (e) d*I/*d*V* map simultaneously acquired with the topographic image (d) (setpoint: *V*_t_ = −170 mV, *I*_t_ = 100 pA), showing high conductance at each edge of the triangular island. (f) IPS signals measured on Bi(111) films of different thicknesses (setpoint: *V*_t_ = −2 V, *I*_t_ = 8.0 nA). Each spectral line is vertically shifted by 0.6 (arb. units). When the film thickness exceeds 3 bilayers, the IPS with *n* = 2 begins to split into two sublevels.

As is known, being a semiconductor substrate, Si can possess an electric field with both radial and perpendicular components. In contrast, Sb as the metallic substrate with ${\epsilon }_r \to \infty $ exhibits electrostatic shielding effects. Therefore, the radial tip electric field component ${E}_{r, {\mathrm{tip}}} \approx 0$ and the field lines are perpendicular to the Sb surface (*E_z_*) according to Equation (3), as shown in Fig. 4a. By adjusting tunneling current to modify the tip-sample distance *d* and the tip electric field strength, we obtained the IPSs at different tunneling currents (*I*_t_ = 50 pA–8.0 nA) on 10 BL Bi(111) thin film shown in Fig. 4b. All the IPS peaks move to higher energy as the tunneling current increases (Fig. 4c), suggesting the Stark shift due to electric field component *E_z_*. However, contrary to the situation in Bi(111)/Si(111) (Fig. 2d), the splitting energy between the *n* = 2/2′ IPS does not increase accordingly (Fig. 4d), which indicates that the splitting of IPSs is unrelated to *E_z_*. Therefore, we infer that the increasing splitting energy on Bi(111)/Si(111) is caused by the radial component of the tip electric field. Furthermore, the splitting energy of IPSs on Bi(111)/Sb(111) is approximately 0.3 eV, which is comparable to the smallest splitting energy in Bi(111)/Si(111), suggesting that this value originates from transient active electric fields generated by field-emitted electrons. We further extracted the FWHM of the *n* = 3 and *n* = 4 IPSs, as shown in Fig. S2. The FWHM evolution shows a substrate- and current-dependent trend similar to that of the *n* = 2 splitting energy. On Bi(111)/Si(111), where the radial field is less effectively screened, both the *n* = 2 splitting and the higher-*n* FWHM evolve strongly with tunneling current. On Bi(111)/Sb(111), both effects are much weaker. This correlation suggests that higher IPS levels are split by the same field-induced response, although their individual sublevels are not spectroscopically resolved.

**Figure 4.**
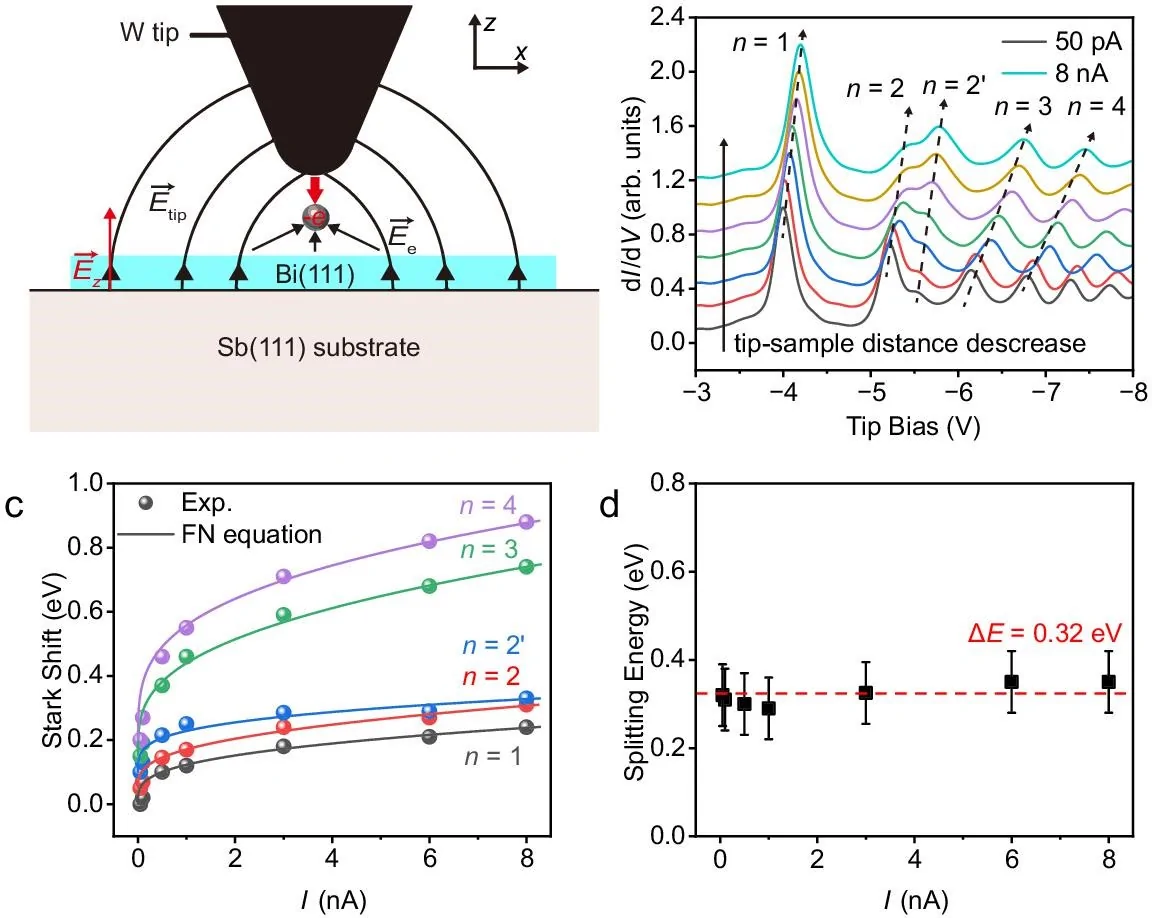
Electric field dependence of IPSs on Bi(111)/Sb(111). (a) Schematic diagram of the electric field distribution in a metal–vacuum–metal tunnel junction. Compared with electric field distribution on semiconductor substrates, the more metallic Sb substrate shields the radial active electric field *E_r_*. Since field emission of electrons is an ultrafast process that cannot establish electrostatic equilibrium with the substrate, the active electric field of the electrons cannot be effectively blocked. (b) IPSs recorded at different setpoints (*V*_t_ = −2 V, *I*_t_ = 50 pA–8.0 nA). Each spectral line is vertically shifted by 0.1 (arb. units). (c) Energy shift of IPSs at different tunneling currents due to the Stark effect. The points were fitted using the FN equation. (d) The splitting energy ${\mathrm{\Delta }}E = {E}_{n = 2} - {E}_{n = 2^{\prime}}\ $varies with tunneling currents, revealing the splitting remains nearly constant.

In addition to the radial active electric field component, there is another factor leading to the splitting of IPSs: symmetric and antisymmetric IPSs series. For a free-standing 2D material, two Rydberg series are expected to emerge due to the charges on each side of the material (symmetric state *n^+^* and antisymmetric state *n^−^*). In this situation, the most pronounced splitting is generally observed for the *n* = 1 IPS [35,36]. However, the peak splitting occurs at the IPS with *n* = 2 on Bi(111). We also measured the IPS spectra across a step of Bi(111) island shown in Fig. S3, which indicates no obvious site dependence, further ruling out the possibility that this splitting is caused by confinement effects from quantum wells [37] or nanodots [14,38] formed in a film with finite thickness.

## DISCUSSIONS

The results above establish several key experimental trends. First, the *n* = 1 IPS remains unsplit, whereas the *n* = 2 IPS on Bi(111) shows a resolved doublet and the higher-*n* IPS peaks exhibit enhanced linewidths, suggesting contributions from unresolved sublevel structures. Second, the anomalous IPS splitting is not observed in 1–2 BL Bi(111), whereas it emerges once the film thickness exceeds ∼3 BL and remains observable in thicker films. Finally, the current dependence of the splitting is strongly affected by substrate screening. These observations indicate that the anomalous IPS response is not a simple field-emission artifact.

A key question is what degree of freedom of the IPS is lifted to produce the observed splitting. In the conventional one-dimensional image-potential model, IPSs are described as states confined along the surface-normal direction and freely extended in the surface plane. In this simplified picture, each IPS is mainly labeled by a principal quantum number *n*, and no additional sublevel structure is expected within a given *n* level. However, in the STM junction, the field-emitted electron and its image charge generate a laterally nonuniform attractive potential centered below the tip. The lateral part of this potential can be approximately written as ${V}_{xy} \propto - {( {{d}^2 + {r}^2} )}^{ - 1/2}$, where *r* is the lateral distance from the tip apex and *d* is the tip-sample distance. Such a center-symmetric lateral potential provides an additional orbital-like degree of freedom and allows IPSs to be discussed, by analogy with a hydrogen-like bound-state problem, in terms of orbital angular momentum quantum numbers *l* in addition to the principal quantum number *n*. In this picture, the *n* = 1 IPS contains only the lowest orbital-like component and therefore does not show a resolved splitting. The *n* = 2 IPS is the lowest state for which different orbital-like components can be spectroscopically separated, giving rise to the observed *n* = 2/2′ doublet. For higher-*n* IPSs, more sublevel components are expected. However, their individual separations can become comparable to or smaller than the intrinsic lifetime broadening, field-induced broadening, and spectral overlap of IPSs. As a result, these higher-*n* sublevels are not resolved as distinct peaks but merge into broader and more asymmetric spectral envelopes. The anomalous field-dependent broadening of the higher-*n* IPS peaks is consistent with this unresolved-sublevel interpretation.

The thickness dependence indicates that developed Bi(111) surface electronic states are required for this response. It has been demonstrated that single-bilayer Bi(111) is a 2D TI [39], whereas bulk Bi(111) manifests as a 3D HOTI [18,19,21]. Density functional theory (DFT) calculations also suggest that topological surface states on Bi(111) emerge starting from three bilayers [40]. Such thickness dependence of topological surface states also occurs in other 3D TIs, such as Bi₂Te₃ [41] and Bi₂Se₃ [42]. As the film thickness decreases, the wave functions of the upper and lower surface states overlap spatially, thereby closing the energy gap or inducing a topological phase transition due to the interlayer coupling. The critical thickness at which the topological surface states emerge coincides with the onset of IPS splitting, suggesting that it should correlate with the developed surface states. A particularly relevant alternative is hybridization or level repulsion between an IPS and an underlying Bi(111) surface state. In a simple two-level model:


(5) \begin{eqnarray*}
H = \left[ {\begin{array}{@{}*{2}{c}@{}} {{E}_{{\mathrm{IPS}}}}&\quad V\\
V&\quad {{E}_{\mathrm{s}}} \end{array}} \right],
\end{eqnarray*}

the coupled energies can be written as ${E}_ \pm = \frac{1}{2}( {{E}_{{\mathrm{IPS}}} + {E}_s} ) \pm \frac{1}{2}\sqrt {{{( {{E}_{{\mathrm{IPS}}} - {E}_{\mathrm{s}}} )}}^2 + 4{V}^2\ } $, where ${E}_{{\mathrm{IPS}}}$ is the Stark-shifted IPS energy, ${E}_{\mathrm{s}}$ is the energy of the underlying surface electronic state, and *V* is the coupling strength between them. In this model, the splitting energy is ${\mathrm{\Delta }}E = \sqrt {{{( {{E}_{{\mathrm{IPS}}} - {E}_{\mathrm{s}}} )}}^2 + 4{V}^2\ } $. Thus, the splitting should be governed mainly by the detuning between the IPS and the surface state. If the observed splitting originated from a simple avoided crossing with a fixed surface state, the Stark shift of the IPS should modify the splitting in both substrate systems. However, in our experiments, the metallic-substrate case shows pronounced Stark shifts without a corresponding increase of the splitting (Fig. 4d). In comparison, no comparable IPS splitting has been reported on conventional metal surfaces with Shockley surface states (Cu(111)) [43], Rashba-type surface states (Au(111)) [44], conventional first-order TIs (Bi_2_Te_3_) [15] or the topological semimetal (Sb(111)) [16]. Therefore, the observed anomalous splitting in IPSs is unlikely to arise from trivial surface states alone but may instead be related to the distinctive topological surface state of Bi(111), including its HOTI character.

In the STM field-emission geometry, the field-emitted electrons generate not only a vertical electric field responsible for the conventional Stark shift, but also an active radial electric-field component near the surface. The observed splitting should arise from a perturbation that couples this active electric-field geometry to the orbital-like sublevels of the IPS manifold. Since simple IPS–surface-state hybridization does not naturally account for the combined substrate and current dependences, we further consider the possibility of an effective magnetic-response-like perturbation induced by the active radial electric field and associated with the developed Bi(111) surface state [8].

According to the tip electric field distribution in a vacuum–TI–semiconductor heterojunction (Fig. 5c), the electric field distribution on the TI surface can be regarded as an active electric field *E_r_* with central symmetry in the *x–y* plane, as illustrated in Fig. 5d and e. In a TME picture, an active electric field generates Hall currents ${j}_\theta $ centered around the charge. The effective magnetic-response field generated by all current loops can be written as


(6) \begin{eqnarray*}
{B}_z \propto \frac{1}{{{\epsilon }_r + 1}}\frac{1}{{{{\left( {z + d} \right)}}^2}},
\end{eqnarray*}

where *d* is the tip–sample distance and *z* is the height measured from the surface along the surface-normal direction. If *d* << *z*, an equivalent magnetic field is proportional to ${z}^{ - 2}$ at the center of the Hall circulation, consistent with the active magnetic field from a monopole-like TME effect. If the IPS manifold is described within a hydrogen-like model with orbital degrees of freedom, the monopole-like TME response can lift the orbital degeneracy and split the IPSs into additional sublevels (see Supplementary Materials IV). For simplicity, we interpret the IPS splitting in an effective magnetic field by analogy with the orbital Zeeman effect of a hydrogen-like system. The intrinsic orbital magnetic moment ${\mu }_B$ associated with angular momentum *l* acquires an additional energy contribution under the effective magnetic field, resulting in a splitting energy described by ${\mathrm{\Delta }}{E}_{{\mathrm{split}}} = {\mu }_B{B}_z\sqrt {l( {l + 1} )} $, *l* = 0 … *n −* 1. We performed line-shape fitting of IPSs with different principal quantum numbers *n* by considering the corresponding angular momentum quantum numbers *l*, and the results are presented in Fig. 5a. The estimated energy scale of this effective response is comparable to the experimentally observed *n* = 2/2′ splitting, suggesting that the monopole-like TME scenario provides a plausible phenomenological interpretation of the observed IPS anomaly (see Supplementary Materials V). In contrast to a uniform magnetic field, the monopole-like TME response produces an effective out-of-plane field that decays approximately as ${B}_z\ \sim \ {z}^{ - 2}$. Here, *z* denotes the average distance of the IPS orbital from the sample surface, and the characteristic orbital radius of the IPS level is expected to scale approximately as ${z}_n\ \sim \ {n}^2{z}_1$ [45]. Consequently, the effective splitting of higher-*n* IPSs is reduced and can be easily masked by lifetime broadening and spectral overlap, making their sublevels difficult to resolve as separate peaks.

**Figure 5.**
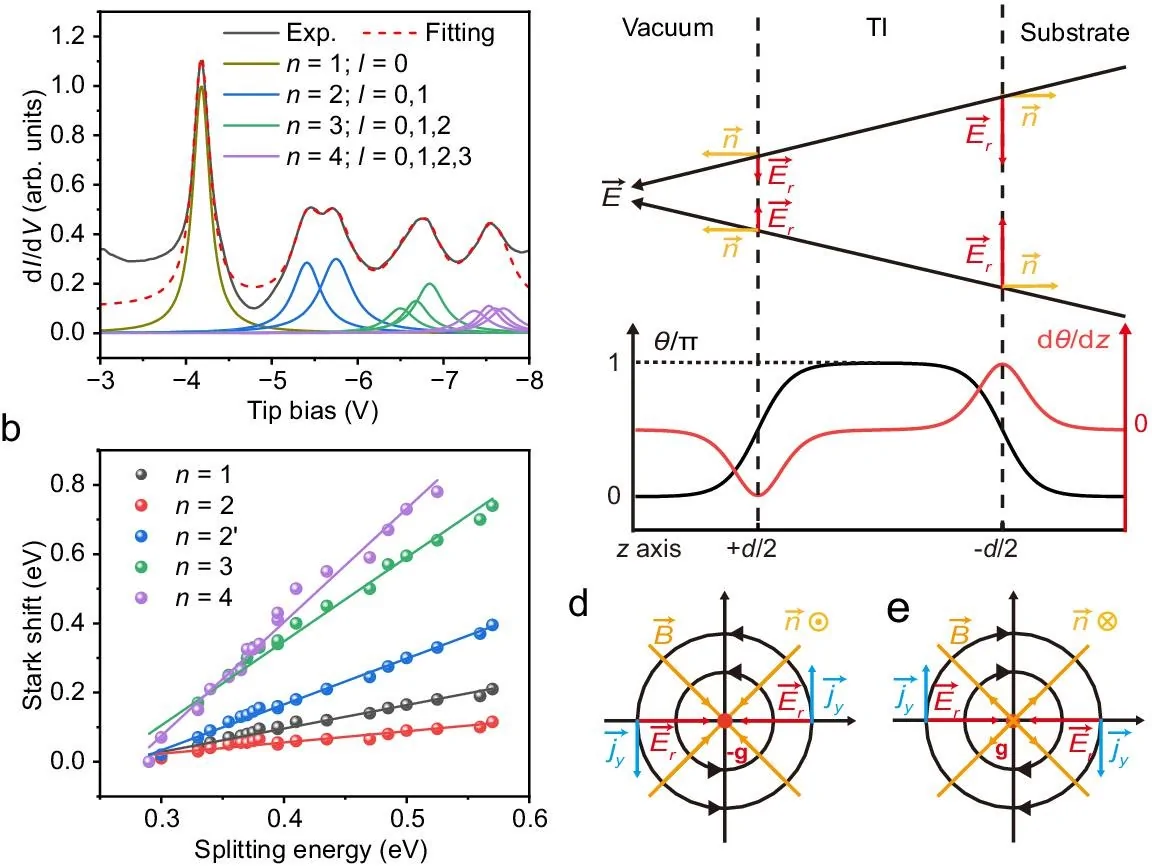
Theoretical model of monopole-like TME response. (a) IPS spectra acquired on the 10 BL Bi(111)/Si(111) surface, refitted by considering the orbital angular momentum quantum number *l*. (b) Linear relationship between the Stark shift of each IPS and the splitting energy between the 2/2′ sublevels. (c) Vacuum–TI–substrate sandwich structure. Vacuum and substrate are topologically trivial (*θ = 0*), and the central TI represents topologically nontrivial ($\theta = {\mathrm{\pi }}$). An applied electric field at the interface between TIs and topologically trivial materials generates a Hall current, which can be expressed as ${{\boldsymbol{J}}}_{{{\boldsymbol {Hall}}}} = ( {{\partial }_{\boldsymbol{z}}{\boldsymbol{\theta }}} ) \times {\boldsymbol{E}}( {\boldsymbol{r}} )$. (d and e) Top view of Hall circulation at two surfaces of the TI (*z = ± d/2*), and equivalent active magnetic field is generated at the center of the circulation.

In field emission mode, the relationship between the tunneling current *J* and electric field *E_z_* can be described by the Fowler–Nordheim (FN) equation $J = \alpha E_z^2{e}^{ - \beta /{E}_Z}$, where the fitting parameters $\alpha ,\ \beta $ are jointly determined by factors such as the tip shape and the work functions of the tip and sample. The emitted electrons and their mirror charges form an electric dipole pair, whose intrinsic dipole moment *p* undergoes a Stark shift under the electric field ${E}_z$. For a pair of opposite charges separated by 1 nm and subjected to an electric field of 10^9^ V m^−1^, the estimated Stark energy shift is ∼1 eV, consistent with the observed magnitude in our experiments.

The interaction between IPSs and the electric field is approximately dominated by the first-order Stark effect, inducing an energy shift of ${\mathrm{\Delta }}{E}_{{\mathrm{stark}}} \propto {p}_n{E}_z \propto {d}^{ - 2}$ (from Equation (3)). Therefore, both the Stark shift of each IPS and the splitting energy between the *n* = 2/2′ IPS sublevels on Bi(111)/Si(111) are expected to exhibit similar dependences on the tip–sample distance. The Stark shift and the splitting energy between the *n* = 2/2′ IPS sublevels are expected to follow the FN dependence on tunneling current, and the fitting results are shown in Fig. 2c and d. We then extracted the Stark shift and splitting energy between *n* = 2/2′ IPS at different tip–sample distances on the Bi(111)/Si(111) surface and plotted them in Fig. 5b, confirming a linear relationship. This correlation indicates that ${\mathrm{\Delta }}{E}_{{\mathrm{split}}} \propto {\mathrm{\Delta }}{E}_{{\mathrm{stark}}} \propto {d}^{ - 2}$, consistent with the mechanism described above and further supports our speculations. Within a phenomenological framework, these observations can be understood in terms of an effective magnetic response associated with a monopole-like TME scenario.

## CONCLUSION

In summary, we observed an anomalous splitting of the IPS in Bi(111) thin films. By tuning the dielectric properties of the substrate and the film thickness, we found that the splitting is strongly correlated with the radial component of the active electric field and with the development of the Bi(111) surface electronic structure. Based on phenomenological models, we propose that the radial electric field may generate an equivalent magnetic field through the TME, which in turn contributes to the splitting of IPS sublevels. More importantly, our results show that IPS spectroscopy naturally provides an experimental route for generating the active radial electric-field geometry envisioned in the Qi model and for probing the resulting surface response spectroscopically. Accordingly, the experimentally established result is the anomalous IPS response and its systematic dependence on film thickness, substrate screening, and tunneling conditions, whereas its connection to a monopole-like TME response remains a phenomenological interpretation requiring further direct verification. Although there are currently no effective experimental methods to detect whether field-emitted electrons actually induce magnetic fields near a TI surface, the anomalous splitting of IPSs may serve as a possible spectroscopic signature compatible with a monopole-like TME scenario. However, further magnetic-response-sensitive or topology-controlled experiments are required to establish the microscopic origin of the monopole-like TME effect.

## METHODS

### Sample preparation

The experiments were performed in a home-built low-temperature STM system equipped with a molecular beam epitaxy chamber, with a base pressure of 3 × 10^−10^ mbar. High-purity Bi (99.999%) was evaporated from a homemade evaporation source. The silicon wafer was *n*-type doped with a resistivity of 0.05 ${\mathrm{\Omega }} \cdot {\mathrm{cm}}$. Si(111) wafer was flashed to about 1200°C several times to obtain a 7 × 7*\ *reconstruction as the substrate. A clean Sb(111) surface was achieved through Ar ion sputtering and then annealing at 400°C for several cycles. The $\sqrt{3} $-Bi reconstruction was prepared by depositing a monolayer of Bi atoms onto*\ *Si(111) at room temperature, followed by annealing at 380°C. Bi(111) films on Si(111) and Sb(111) was prepared by room temperature deposition, at a deposition rate of about 0.3 ML min^−1^.

### STM measurements

STM measurements were performed at liquid-helium temperature with a tungsten tip. STM images were obtained in the constant-current mode. The d*I*/d*V* signals were recorded by standard lock-in detection with a modulation of 20 mV at a frequency of 931 Hz while sweeping the sample bias in an open feedback loop configuration. When probing IPSs, the STM was operated in constant-current mode, where the feedback loop controlled the tip-sample distance *z* to maintain a constant current *I*_t_. All IPS spectra were measured with a bias step of 15 mV, an integration time of 200 ms per point, and a settling time of 20 ms per point.

## Supplementary Material

nwag548_Supplemental_File
