# Supplementary material for: Potential for detecting a monopole-like topological magnetoelectric response using image potential states
Source: Natl Sci Rev. 2026 Aug 26;13(20):nwag548. doi: 10.1093/nsr/nwag548 (PMC13702028; doi:10.1093/nsr/nwag548)
Supplement: Supplementary file 1 [file nwag548_supplemental_file.docx]

Supplemental Materials for

**Potential for detecting a monopole-like topological magnetoelectric response using image potential states**

Yang Zhan^1,2^, Huaiyu Zhang^1,2^, Yu Wang^1,2^, Chenmin Liu^1,2^, Jian-Qiang Zhong^3^, Yeliang Wang^4^, Hong-Jun Gao^1,2^, Yi-Qi Zhang^1,2^, Baojie Feng^1,2^, Peng Cheng^1,2,*^, Lan Chen^1,2,*^

*^1^ Institute of Physics, Chinese Academy of Sciences, Beijing 100190, China.*

*^2^ School of Physical Sciences, University of Chinese Academy of Sciences, Beijing 101408, China*

*^3^ S**chool of Physics, Hangzhou Normal University, Hangzhou, Zhejiang 3111121, China*

*^4^* *MIIT Key Laboratory for Low-Dimensional Quantum Structure and Devices, School of Integrated Circuits and Electronics, Beijing Institute of Technology, Beijing 100081, China*

*Corresponding authors. Emails: lchen@iphy.ac.cn; pcheng@iphy.ac.cn

**The PDF file includes:**

**I. IPSs measured on topologically trivial semiconductor**

**II. FWHM of *n* = 3,4 IPSs**

**III. IPS on Bi(111) island**

**IV. Energy-Level Splitting of IPS Electrons in a Monopole-like Magnetic Potential**

**V.** **Estimation of n=2 IPS splitting energy**

**I.IPSs measured on topologically trivial semiconductor**

**Figure S1.** Image potential states measured on Si(111) and √3-Bi. (a) Topographic image of Si(111)(7$\times$7) surface (200×200nm^2^) and the inset shows atomically resolved STM topographic image (7.5×7.5nm^2^) (b) IPS measured on the surface of a (setpoint: V_t_ = −2V, *I*_t_ = 200pA). (c) Topographic image of √3-Bi surface (200×200nm^2^) and the inset shows atomically resolved STM topographic image (4×4nm^2^) (d) IPS measured on the surface of c (setpoint: V_t_ = −2V, *I*_t_ = 200pA). Both (b) and (d) employ Gaussian functions to fit the IPS peaks for each principal quantum number *n*.

**II. FWHM of *n* = 3,4 IPSs**

**Figure S2.** FWHM of *n* = 3,4 IPSs on Bi(111)/Si(111) and Bi(111)/Sb(111). The films grown on both substrates are 10BL thick. On semiconductor Si substrate as the tunnel junction spacing decreases (tunnel current *I* increases), the full width at half maximum (FWHM) of *n* = 3,4 IPSs also increases correspondingly. On metallic Sb substrates, the FWHM does not depend on the tunnel junction distance. The trend resembles the splitting behavior observed in *n* = 2 IPSs.

**III. IPS on Bi(111) island**

**Figure S3.** Spatial dependence of IPSs across Bi(111) island. (a) 50×50 nm^2^ STM image of a Bi island with the shape of a triangle, with an island area over 150 nm^2^. (b) Height profile along the red arrow in (a). (c) IPS at N+1 BL-Bi(111) (black line) and at the N BL-Bi(111) (red line), the red line is biased by 1.0 (a. u.). (d) 2D visualization of the IPS along the red arrow in (a) ($I_{t}=50pA$).

**IV.** **Energy-Level Splitting of IPS Electrons in a Monopole-like Magnetic Potential**

If an electron placed near the surface of a topological insulator induces a monopole-like topological magnetoelectric response, the IPS electron can be treated as moving in the combined Coulomb-like potential of an image charge and the vector potential of an effective monopole-like magnetic response (Part of the model construction follows the theoretical ideas developed in Refs. [1] and [2].). The Hamiltonian can be written as

$H=\frac{1}{2\mu}\left[ \boldsymbol{p}+e\boldsymbol{A}_{g}\left( r \right) \right]^{2}-\frac{\kappa}{r}$	(S4.1)

where $\mu$ is the effective mass, $e$ is the electron charge, $\boldsymbol{A}_{g}$ is the vector potential generated by the monopole, and $-\kappa/r$ represents the attractive Coulomb-like image potential. In the presence of a magnetic monopole, the canonical momentum $\boldsymbol{p}$ should be replaced by the mechanical momentum

$\boldsymbol{\pi=p+}e\boldsymbol{A}_{\boldsymbol{g}}$	(S4.2)

Accordingly, the orbital angular momentum and orbital magnetic moment should be redefined using the mechanical momentum:

$\boldsymbol{\Lambda}\boldsymbol{=r\times\pi}$	(S4.3)

$\boldsymbol{\mu}_{orb}=-\frac{e}{2m}\left\langle\boldsymbol{\Lambda} \right\rangle$	(S4.4)

However, in a charge-monopole system, the mechanical orbital angular momentum $\boldsymbol{\Lambda}$ alone is not conserved. This can be understood from the classical equation of motion. The central Coulomb force does not produce a torque, whereas the magnetic Lorentz force generated by the monopole field does produce a torque on the electron. As a result, the conserved quantity is not the ordinary orbital angular momentum, but the total angular momentum of the charge-monopole system:

$\boldsymbol{J}=\boldsymbol{\Lambda}-s\hbar\hat{\boldsymbol{r}}\boldsymbol{,}s\boldsymbol{=}\frac{qg}{4\pi\hbar}$	(S4.1)

The second term, $-s\hbar\hat{\boldsymbol{r}}$, can be understood as the angular momentum carried by the electromagnetic field of the charge-monopole system. Therefore, once a monopole-like field is introduced, the angular wave functions are no longer ordinary spherical harmonics. Instead, they become monopole harmonics, and the electron wave function is forced to acquire a nontrivial angular structure. The lowest allowed angular momentum is also modified and must satisfy

$$j\geq\left| s \right|$$

To see how this affects the energy spectrum, we start from the minimal-coupling Hamiltonian

$H_{0}=\frac{\boldsymbol{\pi}^{2}}{2\mu}-\frac{\kappa}{r}$	(S4.6)

and decompose the mechanical momentum in spherical coordinates into radial and angular components

$\boldsymbol{\pi}^{2}=p_{r}^{2}+\frac{\boldsymbol{\Lambda}^{2}}{r^{2}}=p_{r}^{2}+\frac{\boldsymbol{J}^{\boldsymbol{2}}\boldsymbol{-}\boldsymbol{\hbar}^{\boldsymbol{2}}s^{2}}{r^{2}}$	(S4.7)

The effective centrifugal term in the radial equation is then modified from the ordinary hydrogenic form

$$\frac{\hbar^{2}l\left( l+1 \right)}{2\mu r^{2}}$$

to

$l_{eff}=\frac{\hbar^{2}}{2\mu r^{2}}[j\left( j+1 \right)-s^{2}]$	(S4.8)

Therefore, the radial Schrödinger equation becomes

$\{-\frac{\hbar^{2}}{2\mu}\frac{d^{2}}{dr^{2}}+\frac{\hbar^{2}}{2\mu r^{2}}\left[ j\left( j+1 \right)-s^{2} \right]-\frac{\kappa}{r}\}u_{n_{r}j}\left( r \right)=Eu_{n_{r}j}\left( r \right)$	(S4.9)

Compared with the ordinary hydrogen atom, the angular-momentum term $l(l+1)$ is replaced by

$$j\left( j+1 \right)-s^{2}$$

Solving the radial equation gives the energy levels

$E_{n_{r}j}=-\frac{\mu\kappa^{2}}{2\hbar^{2}}\frac{1}{\nu_{n_{r}j}^{2}}, \nu_{n_{r}j}=n_{r}+\frac{1}{2}+\sqrt{\left( j+\frac{1}{2} \right)^{2}-s^{2}}$	(S4.10)

For the ordinary hydrogenic case with s = 0, the energy depends only on the principal quantum number, leading to the usual degeneracy. In contrast, for a monopole system with $s\neq0$, the energy depends on both $n_{r}$and $j$. Thus, the introduction of a magnetic monopole lifts the degeneracy of the electron-dyon energy levels.

| *s* = 0 | | | *s* =1/2 | | |
| --- | --- | --- | --- | --- | --- |
| $n_{r}$ | l | n | $n_{r}$ | j | $\nu_{n_{r}j}$ |
| 0 | 0 | 1 | 0 | 1/2 | 1.366 |
| 0 | 1 | 2  （degenerate） | 0 | 3/2 | 2.437 |
| 1 | 0 |  | 1 | 1/2 | 2.366 |
| 0 | 2 | 3  （degenerate） | 0 | 5/2 | 3.458 |
| 1 | 1 |  | 1 | 3/2 | 3.436 |
| 2 | 0 |  | 2 | 1/2 | 3.366 |

Table I. Comparison of the quantum numbers and effective principal quantum numbers for a hydrogen-like atom (*s* = 0) and an electron–dyon system (*s* = 1/2). The hydrogen-like energy levels with the same principal quantum number *n* are degenerate, whereas the introduction of a magnetic monopole lifts this degeneracy and leads to different effective quantum numbers $\nu_{n_{r}j}$.

**V.** **Estimation of n=2 IPS splitting energy**

The IPSs has a large orbital radius, and we can equate the magnetic field felt by an electron with a uniform magnetic field, where the strength of the magnetic field felt by the electron is proportional to the magnitude of the magnetic monopole $g$ and is inversely proportional to the square of the orbital radius $r$ (Gaussian units):

$B=\frac{g}{r^{2}}\boldsymbol{e}_{\boldsymbol{r}}$	(S5.1)

The energy attached to the orbital magnetic moment in a uniform magnetic field can be expressed as:

$\Delta E(n)=\mu_{l}B=\frac{eg\hbar}{2\mu c}\frac{\sqrt{l\left( l+1 \right)}}{r^{2}}$	(S5.2)

where u is the electron effective mass and c is the speed of light. For hydrogen-like atoms, the electrons will be distributed around the nucleus as an electron cloud, so we should replace the magnetic field with the average of the magnetic field $\left\langle B \right\rangle$ felt by the electrons：

$\Delta E(n)=\mu_{l}\left\langle B \right\rangle=\frac{eg\hbar\sqrt{l\left( l+1 \right)}}{2\mu c}\left\langle\frac{1}{r^{2}} \right\rangle$	(S5.3)

The Hamiltonian of the radial equation for the electron in the central potential field can be expressed as:

$\hat{H}=\frac{\hbar^{2}}{2\mu}\frac{d^{2}}{dr^{2}}+V\left( r \right)+\frac{l\left( l+1 \right)\hbar^{2}}{2\mu r^{2}}$	(S5.4)

According to Hellmann-Feynman theorem:

$\frac{\partial E_{n}}{\partial\lambda}=\left\langle\frac{\partial\hat{H}}{\partial\lambda} \right\rangle$	(S5.5)

Consider *l* as a parameter:

$\left\langle\frac{\partial\hat{H}}{\partial l} \right\rangle=\left( l+\frac{1}{2} \right)\frac{\hbar^{2}}{\mu}\left\langle\frac{1}{r^{2}} \right\rangle=\frac{\partial E_{n}}{\partial l}=\frac{\partial E_{n}}{\partial n}=\frac{e^{2}}{n^{3}a_{1}}$	(S5.6)

where $a_{1}$ is Bohr radius. And we can get:

$\left\langle\frac{1}{r^{2}} \right\rangle=\frac{1}{a_{1}^{2}}\frac{1}{\left( l+1/2 \right)n^{3}}$	(S5.7)

Combining equations S4.1 and S4.2, we get:

$\Delta E(n)=\frac{eg\hbar}{2\mu ca_{1}^{2}}\frac{\sqrt{l\left( l+1 \right)}}{\left( l+1/2 \right)n^{3}}$	(S5.8)

For each principal quantum number n, the orbital angular momentum quantum number can take the value $l = 0,\ldots, n-1$. The energy difference between $l = 0$ and $l = n-1$ can be written as:

$\Delta E(n)=\frac{eg\hbar}{2\mu ca_{1}^{2}}\frac{\sqrt{n\left( n-1 \right)}}{n^{3}(n-1/2)}$	(S5.9)

Disregarding the Stark effect, the intrinsic IPS energy levels on graphene are:

$E_{n}=E_{vac}-\frac{0.85}{\left( n+a \right)^{2}}$	(S5.10)

where $a$ is a correction factor of the crystal field^5^, the first Rydberg energy level is $E_{1}=-0.85 eV$, and it can also be described as:

$E_{1}=-\frac{\mu e^{4}}{2\hbar^{2}}$	(S5.11)

And the hydrogen atom ground state:

$E_{1,H}=-\frac{m_{e}e^{4}}{2\hbar^{2}}=-13.6eV$	(S5.12)

It can be obtained that for the IPS state the effective mass of the electron is $\mu=0.0625m_{e}$, the orbital radius of the first three IPS state is about:

$a_{1}=\frac{\hbar^{2}}{\mu e^{2}}=8.5\times{10}^{-8} cm$	(S5.13)

If the electron-dyon quasiparticle is considered to be both in a vacuum environment, the electric charge carried by the double charge is related to the magnetic charge as：

$g=\frac{2q}{\alpha}$	(S5.14)

where $\alpha$ is fine Structure Constants. The splitting size of n=2 IPS can be expressed as

$\Delta E(n)=\mu_{l}B=\frac{e^{2}\hbar}{\alpha\mu ca_{1}^{2}}\frac{\sqrt{n\left( n-1 \right)}}{n^{3}(n-1/2)}=3.31\times{10}^{-13}erg=204meV$	(S5.15)

**Reference:**

1. Xiao SF, Liu QH. Geometric momentum and angular momentum for charge monopole system. *Modern Phys Lett A.*2018; **33**:1850125.
2. Chen JL, Ge ML, Xue K. The monopole-hydrogen atom system and its connection with a four-dimensional harmonic oscillator *J. Phys. A: Math. Gen.*1999; **32**, 945–53.
